# Reduced phosphatidylcholine synthesis suppresses the embryonic lethality of seipin deficiency

**DOI:** 10.1093/lifemeta/loac021

**Published:** 2022-09-08

**Authors:** Jinglin Zhu, Sin Man Lam, Leilei Yang, Jingjing Liang, Mei Ding, Guanghou Shui, Xun Huang

**Affiliations:** State Key Laboratory of Molecular Developmental Biology, Institute of Genetics and Developmental Biology, Chinese Academy of Sciences, Beijing 100101, China; University of Chinese Academy of Sciences, Beijing 100049, China; State Key Laboratory of Molecular Developmental Biology, Institute of Genetics and Developmental Biology, Chinese Academy of Sciences, Beijing 100101, China; State Key Laboratory of Molecular Developmental Biology, Institute of Genetics and Developmental Biology, Chinese Academy of Sciences, Beijing 100101, China; State Key Laboratory of Molecular Developmental Biology, Institute of Genetics and Developmental Biology, Chinese Academy of Sciences, Beijing 100101, China; State Key Laboratory of Molecular Developmental Biology, Institute of Genetics and Developmental Biology, Chinese Academy of Sciences, Beijing 100101, China; University of Chinese Academy of Sciences, Beijing 100049, China; State Key Laboratory of Molecular Developmental Biology, Institute of Genetics and Developmental Biology, Chinese Academy of Sciences, Beijing 100101, China; University of Chinese Academy of Sciences, Beijing 100049, China; State Key Laboratory of Molecular Developmental Biology, Institute of Genetics and Developmental Biology, Chinese Academy of Sciences, Beijing 100101, China; University of Chinese Academy of Sciences, Beijing 100049, China

**Keywords:** seipin, phosphatidylcholine, embryogenesis, lipid droplet

## Abstract

Seipin plays a vital role in lipid droplet homeostasis, and its deficiency causes congenital generalized lipodystrophy type II in humans. It is not known whether the physiological defects are all caused by cellular lipid droplet defects. Loss-of-function mutation of *seip-1*, the *Caenorhabditis elegans* seipin ortholog, causes embryonic lethality and lipid droplet abnormality. We uncover *nhr-114* and *spin-4* as two suppressors of *seip-1* embryonic lethality. Mechanistically, *nhr-114* and *spin-4* act in the “B12-one-carbon cycle-phosphatidylcholine (PC)” axis, and reducing PC synthesis suppresses the embryonic lethality of *seip-1* mutants. Conversely, PC deficiency enhances the lipid droplet abnormality of *seip-1* mutants. The suppression of *seip-1* embryonic lethality by PC reduction requires polyunsaturated fatty acid. In addition, the suppression is enhanced by the knockdown of phospholipid scramblase *epg-3*. Therefore, seipin and PC exhibit opposite actions in embryogenesis, while they function similarly in lipid droplet homeostasis. Our results demonstrate that seipin-mediated embryogenesis is independent of lipid droplet homeostasis.

## Introduction

Lipids are essential for life and take part in nearly all physiological processes. Abnormal lipid metabolism is associated with many diseases, including developmental, neuronal, and reproductive diseases, as well as metabolic syndromes. Regulation at both the cellular and tissue levels is required to maintain the organismal homeostasis of lipid metabolism. At the cellular level, lipid droplets, which originate from the endoplasmic reticulum (ER), are hub organelles for neutral lipid storage and utilization [1, 2].

Seipin, an integral ER protein, plays an important role in lipid droplet homeostasis [3, 4]. Located at the contact site between the ER and the budding nascent lipid droplet, seipin possesses a luminal lipid-binding motif between its two transmembrane domains and forms oligomers [5, 6]. Seipin stabilizes nascent lipid droplets and promotes their growth by facilitating the transfer of neutral lipid from the ER into the associated lipid droplets. In seipin-deficient cells, abnormal partitioning of neutral lipids results in the formation of tiny lipid droplets and some supersized lipid droplets [7, 8]. Besides seipin, other proteins or lipid factors, including FIT, Snx14, ACSL3, and phosphatidylcholine (PC), have also been identified to promote lipid droplet biogenesis and/or lipid droplet growth [9–13].

In addition to the cellular lipid droplet defect, seipin deficiency also causes Bernardinelli-Seip congenital lipodystrophy 2 (BSCL2)/congenital generalized lipodystrophy type II (CGL2) in humans [14]. The patients lose nearly all their subcutaneous fat tissue and develop many metabolic syndromes, including fatty liver, diabetes, and hypertriglyceridemia, as well as many nonmetabolic disorders, such as muscular hypertrophy, mental retardation, and sperm abnormality [15, 16]. It is not known how seipin deficiency causes so many physiological defects. In particular, it is unclear whether all these defects are due to abnormal lipid droplet homeostasis.

Seipin is conserved from yeast to human. Similar to BSCL2, numerous physiological defects have been reported in *seipin*-deficient animal models [16–20]. *seip-1*, the only ortholog of human seipin in *Caenorhabditis elegans (C. elegans)*, regulates the homeostasis of intestinal lipid droplets and also embryogenesis. Here, through identification and characterization of the *seip-1* suppressors, we found that reducing PC synthesis enhances the lipid droplet defect of *seip-1* mutants, while suppresses the embryonic lethality. The suppression of *seip-1* embryonic lethality by PC reduction requires polyunsaturated fatty acid (PUFA). Therefore, this suggests that seipin-mediated embryogenesis is independent of lipid droplet homeostasis.

## Results

### Deletion of *seip-1* results in embryonic lethality

**Figure 1:**
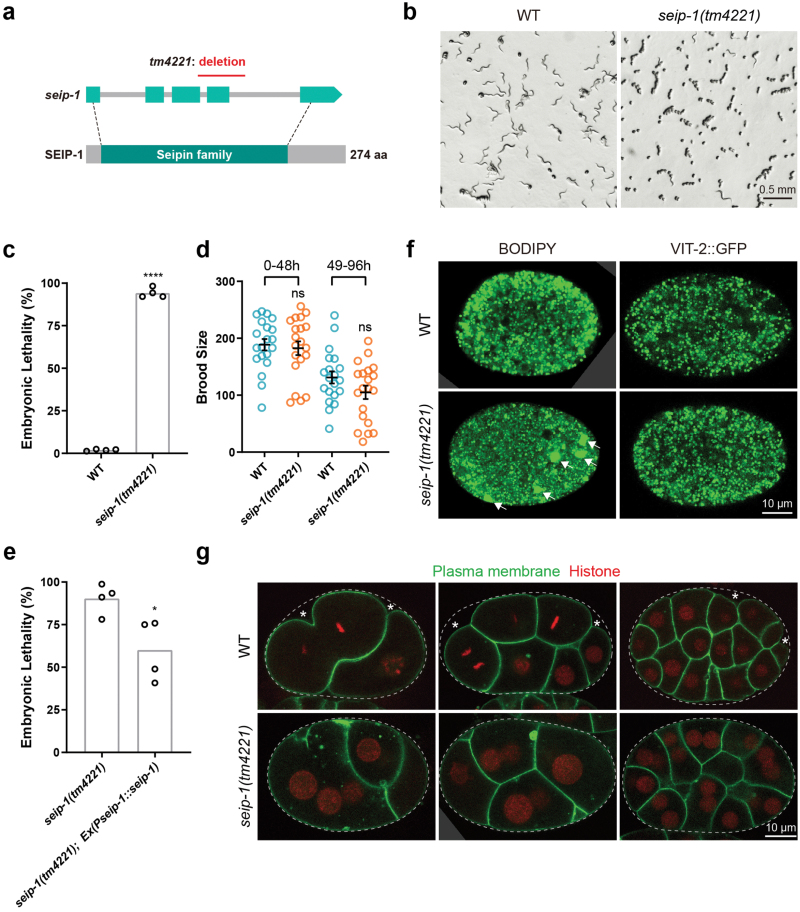
*seip-1(tm4221)* mutants exhibit embryonic lethality and have large lipid droplets. (a) Diagram of the open reading frame and the conserved protein domain of *C. elegans* seipin. Light cyan rectangles indicate the exons and gray lines indicate the introns in the *seip-1* gene. The dark cyan rectangle highlights the conserved region in the SEIP-1 protein. Dashed lines indicate the start and end of the region that encodes the conserved protein domain. The red line indicates the deleted genomic region in the *seip-1(tm4221)* mutant. (b) Bright-field images of worm plates containing wild type and *seip-1(tm4221)* mutant animals. There are many dead embryos and few larvae on the *seip-1(tm4221)* mutant plate. (c) Quantification of embryonic lethality of wild type and *seip-1(tm4221)* mutants. Each point represents a biological repeat. Statistical significance was determined by two-tailed unpaired *t*-test with Welch’s correction, ****P < 0.0001. (d) Quantification of brood size of wild type and *seip-1(tm4221)* mutants over two time periods from the beginning of adulthood. Each point represents a biological repeat. Statistical significances were determined by ordinary one-way ANOVA with *post hoc* Sidak’s test, ns, not significant. (e) Expression of wild-type SEIP-1 in the *seip-1(tm4221)* mutants decreases the embryonic lethality. Each point represents a biological repeat. Statistical significance was determined by two-tailed and unpaired *t*-test, *P < 0.05. (f) Confocal images of embryonic lipid droplets and yolk particles. Lipid droplets were stained with BODIPY and yolk particles were labeled by GFP-tagged lipoprotein VIT-2. Supersized lipid droplets in *seip-1(tm4221)* embryos are indicated by arrows. (g) Confocal images of wild type and *seip-1(tm4221)* embryos with the plasma membrane labeled by GFP::PH(PLC1delta1) and the nucleus labeled by mCherry::Histone. Eggshells are indicated by dashed lines and gaps between eggshells and plasma membranes are indicated by asterisks.

*tm4221*, a deletion allele of *seip-1* with a 299-bp deletion including exon 4 and part of exon 3, was obtained from the *C. elegans* Gene Knockout Consortium (Fig. 1a). We found that *seip-1(tm4221)* mutants exhibited highly penetrant embryonic lethality, whereas brood size was not significantly changed (Fig. 1b–d). An extrachromosomal array expressing the wild-type *seip-1* gene partially rescued the embryonic lethality of *seip-1(tm4221)* mutants (Fig. 1e). These observations suggest that SEIP-1 is critical for *C. elegans* embryonic development.

Since the well-known function of seipin is to control lipid droplet homeostasis, we examined lipid droplets in embryos by BODIPY staining. Compared to wild-type embryos, there were several supersized lipid droplets in *seip-1(tm4221)* mutant embryos (Fig. 1f). In contrast, there was no difference in yolk particles, labeled by the yolk protein VIT-2, between *seip-1(tm4221)* embryos and wild-type embryos. These results indicate that *seip-1(tm4221)* affects lipid droplet homeostasis.

To further characterize the phenotype of *seip-1(tm4221)* embryos, we labeled the plasma membrane and nucleus with fluorescent reporters. We found that *seip-1(tm4221)* embryos often contained multinucleate cells even at the early stage of embryogenesis (Fig. 1g). To understand how these multinucleate cells formed, we used time-lapse microscopy to track cytokinesis. In *seip-1(tm4221)* embryos, cytokinesis proceeded more slowly and often aborted halfway through. Abrupt disruption of the adjoining plasma membrane, resulting in cell fusion, was also found in *seip-1(tm4221)* mutants (Supplementary Fig. S1a). In addition, the plasma membrane of cells in *seip-1(tm4221)* embryos was very close to the supporting outer eggshell, while there was a gap between the plasma membrane and the eggshell in wild-type embryos (Fig. 1g). The eggshell is composed of six layers, among which the rigid chitin layer shapes the embryo and the permeability barrier layer maintains the internal osmotic pressure [21, 22] (Supplementary Fig. S1b). The loss of space between the chitin layer and the plasma membrane in *seip-1(tm4221)* embryos indicates that the permeability barrier was disturbed, which caused hypo-osmotic swelling of the cells. Indeed, electron microscopy showed that the permeability barrier layer was missing in *seip-1(tm4221)* eggshells (Supplementary Fig. S1c). Accordingly, *seip-1(tm4221)* embryos swelled in hypotonic buffer and shrank in hypertonic buffer (Supplementary Fig. S1d). Moreover, the *seip-1(tm4221)* eggshell was permeable to DAPI dye (Supplementary Fig. S1e). These results suggest that *seip-1(tm4221)* embryos have an eggshell permeability defect, consistent with a previous report [18].

### *spin-4* and *nhr-114* suppress the embryonic lethality of *seip-1* mutants

**Figure 2:**
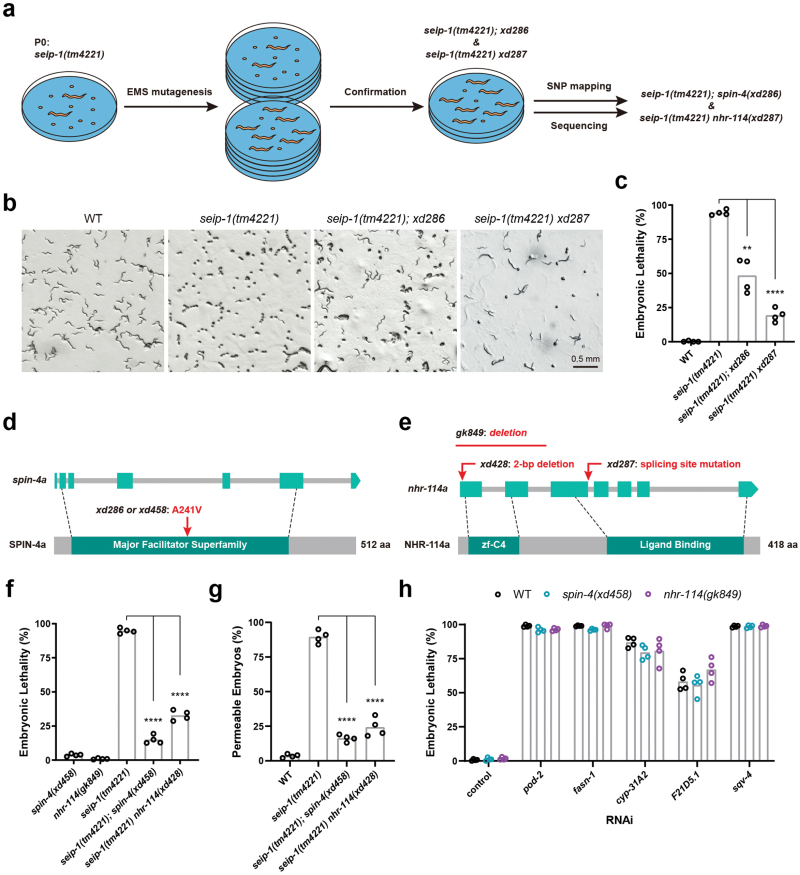
*spin-4* and *nhr-114* mutations suppress the embryonic lethality of the *seip-1* mutants. (a) Workflow for the EMS mutagenesis screen and the identification of suppressor genes. The embryonic lethality phenotype was manifested by contrasting numbers of embryos and larvae on each plate. (b) Bright-field images of worm plates containing wild type, *seip-1(tm4221)*, *seip-1(tm4221);xd286*, and *seip-1(tm4221) xd287* animals. (c) Quantification of the embryonic lethality of wild type, *seip-1(tm4221)*, *seip-1(tm4221);xd286*, and *seip-1(tm4221) xd287*. Each point represents a biological repeat. Statistical significances were determined by Brown-Forsythe and Welch ANOVA with *post hoc* Dunnett’s T3 test, **P < 0.01, ****P < 0.0001. (d) Diagram of the amino acid change in SPIN-4 isoform a caused by the *spin-4(xd286)* and *spin-4(xd458)* mutations. Light cyan rectangles indicate the exons and gray bars indicate the introns in the *spin-4* gene. The dark cyan rectangle highlights the conserved region of the major facilitator superfamily (MFS) in the SPIN-4 protein. Dashed lines indicate the start and end of the region encoding the conserved MFS domain. (e) Diagram of the molecular changes caused by the *nhr-114(xd287)*, *nhr-114(xd428)*, and *nhr-114(gk849)* mutations. Light cyan rectangles indicate the exons and gray lines indicate the introns in the *nhr-114* gene. Dark cyan rectangles highlight the DNA binding (zf-C4) and ligand binding domains in the NHR-114 protein. Dashed lines indicate the start and end of the regions encoding the two conserved domains. (f) Quantification of the embryonic lethality of mutant alleles. Each point represents a biological repeat. Statistical significances were determined by ordinary one-way ANOVA with *post hoc* Dunnett’s test, ****P < 0.0001. (g) Quantification of the permeability of wild type and mutant eggshells. The eggshell was judged to be permeable if the embryo shrunk in a hyperosmotic solution. Each point represents a biological repeat. Statistical significances were determined by ordinary one-way ANOVA with *post hoc* Dunnett’s test, ****P < 0.0001. (h) Quantification of the embryonic lethality of wild type, *spin-4(xd458)* and *nhr-114(gk849)* animals fed on different RNAi bacteria.

To understand how *seip-1* affects eggshell integrity and embryogenesis, we carried out a genetic screen to identify suppressors of the highly penetrant embryonic lethal phenotype in *seip-1(tm4221)* mutants (Fig. 2a). About 2300 genomes were mutagenized by ethyl methanesulfonate (EMS). We identified two suppressors, *xd286* and *xd287*, which partially suppressed the embryonic lethality of *seip-1(tm4221)* mutants (Fig. 2b and c). Single nucleotide polymorphism mapping and whole-genome sequencing identified a missense mutation in *spin-4(xd286)* and a splicing site mutation in *nhr-114(xd287)* (Fig. 2d and e). *spin-4* encodes a lysosomal/late-endosome transmembrane transporter of the major facilitator superfamily, and its human ortholog SPNS1 may export sugar/sphingolipid [23]. *nhr-114* encodes a transcription factor of the nuclear hormone receptor family and acts in the “B12-one-carbon cycle-PC (phosphatidylcholine)” axis [24].

To rule out the possibility that the suppression is caused by other background mutations, we generated *spin-4(xd458)* and *nhr-114(xd428)* mutations by CRISPR-Cas9. These mutations also partially suppressed the embryonic lethality of *seip-1(tm4221)* mutants (Fig. 2f), which demonstrates that *spin-4* and *nhr-114* are *bona fide* genetic suppressors of *seip-1(tm4221)* embryonic lethality. Besides, *spin-4(xd458)* and *nhr-114(xd428)* also suppressed the permeable eggshell defect to a similar extent in *seip-1(tm4221)* embryos (Fig. 2g), which indicates the importance of eggshell integrity to embryonic survival.

It was reported that seipin broadly affects the cellular metabolism of lipids. Previous studies identified several genes involved in lipid or carbohydrate metabolism that are important for the formation of the eggshell, and deficiencies of these genes cause embryonic lethality similar to the *seip-1(tm4221)* mutation [21, 22]. Therefore, we tested the specificity of the suppression of *spin-4* and *nhr-114* mutations in *seip-1(tm4221)* mutants. Because *nhr-114* is closely linked to *seip-1*, we did not obtain a strain carrying the *nhr-114(xd428)* mutation alone. Instead, we used the deletion allele *nhr-114(gk849)* (Fig. 2e). *spin-4(xd458)* and *nhr-114(gk849)* did not suppress the embryonic lethality induced by RNAi of genes involved in fatty acid synthesis (*pod-2* and *fasn-1*), fatty acid modification (*cyp-31A2*), or monosaccharide modification (*F21D5.1* and *sqv-4*) (Fig. 2h). Put together, these results indicate that the *spin-4* and *nhr-114* mutations act as specific suppressors of the embryonic lethality of *seip-1(tm4221)* mutants.

### *spin-4* and *nhr-114* act in the same “B12-one-carbon cycle-PC” pathway

**Figure 3:**
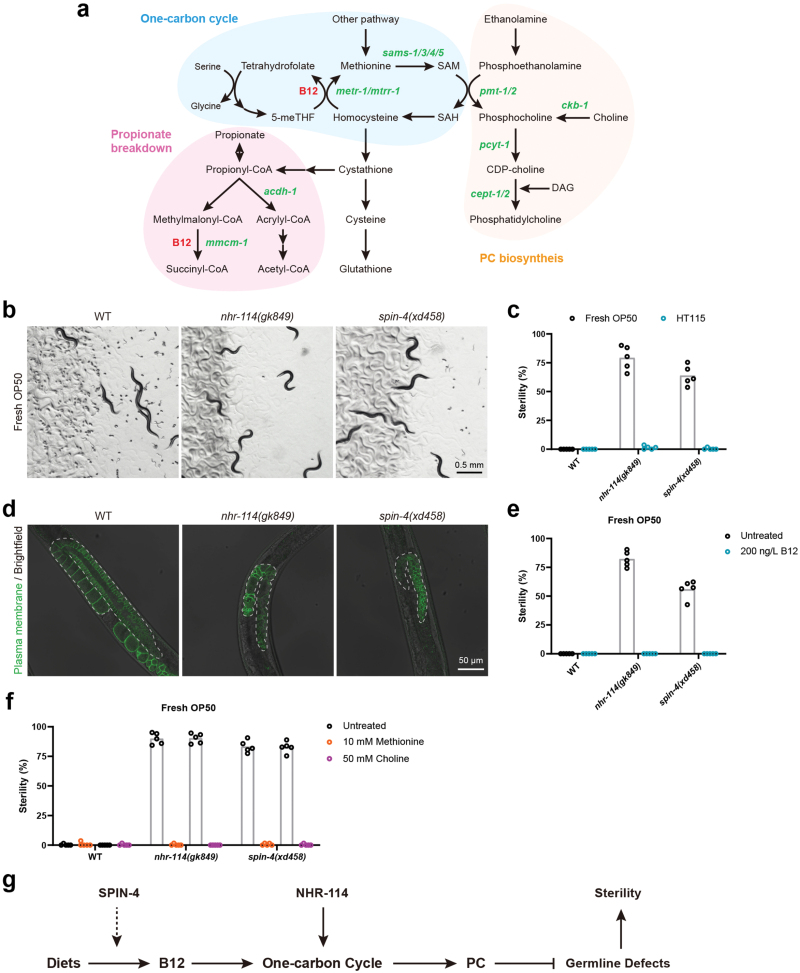
*spin-4* and *nhr-114* act in the “B12-one-carbon cycle-PC” axis. (a) Diagram of the association between the three metabolic pathways: propionate breakdown, one-carbon cycle and PC synthesis. CDP, cytidine 5ʹ-diphosphocholine; 5-meTHF, 5-methyltetrahydrofolate; SAH, S-adenosylhomocysteine; SAM, S-adenosylmethionine; DAG, diacylglycerol. (b) Bright-field images of worm plates carrying wild type, *nhr-114(gk849)* mutants and *spin-4(xd458)* mutants on fresh OP50. Both mutants have a greatly decreased number of offspring (embryos and larvae). (c) Quantification of the sterility of wild type, *nhr-114(gk849)* mutants and *spin-4(xd458)* mutants grown on fresh OP50 or normal HT115 plates. (d) Confocal images of the germlines of day 1 adult wild type, *nhr-114(gk849)* mutants and *spin-4(xd458)* mutants labeled by GFP::PH(PLC1delta1). Dashed lines indicate the germline contours. (e and f) Quantification of the sterility of wild type, *nhr-114(gk849)* mutants and *spin-4(xd458)* mutants cultured on fresh OP50 supplied with or without 200 ng/L B12 (e), 10 mmol/L methionine or 50 mmol/L choline (f). (g) Model of NHR-114 and SPIN-4 acting in the “B12-one-carbon cycle-PC” axis to regulate fertility.

We then investigated how *spin-4* and *nhr-114* suppress the embryonic lethality of *seip-1(tm4221)* mutants. NHR-114 acts in the “B12-one-carbon cycle-PC” pathway [24] (Fig. 3a). As a coenzyme, vitamin B12 regulates two important metabolic reactions in this axis [24] (Fig. 3a). One reaction converts methylmalonyl-CoA to succinyl-CoA in the major propionate breakdown pathway. The second reaction, which is part of the one-carbon cycle, produces S-adenosylmethionine (SAM). SAM acts as a methyl donor to dozens of methyl receptors, including phosphoethanolamine, which is important for the synthesis of PC. When B12 is deficient, NHR-114 activates the expression of several genes in the B12 transport and one-carbon cycle pathway and promotes PC biosynthesis [24]. *nhr-114* mutants displayed a diet-dependent sterility: they are sterile when growing on *Escherichia coli* OP50, while they are fertile when growing on *E. coli* HT115 [25]. *E. coli* HT115 provides more B12 to *C. elegans* than *E. coli* OP50 [26, 27], which explains the diet-dependent sterility phenotype of *nhr-114* mutants.

The link between *spin-4* and *nhr-114* was previously unknown. We observed a similar semisterile phenotype when growing *nhr-114(gk849)* and *spin-4(xd458)* mutants on an OP50 diet (Supplementary Fig. S2a). Notably, the sterility is greatly increased when *nhr-114(gk849)* and *spin-4(xd458)* mutants were fed on a fresh OP50 diet (Fig. 3b and c). Similar to *nhr-114(gk849)*, *spin-4(xd458)* mutants are fully fertile when grown on an HT115 diet (Fig. 3c). In addition, the germlines of *nhr-114(gk849)* and *spin-4(xd458)* mutants were distorted and smaller compared to wild type (Fig. 3d). Taking the suppression of the *seip-1(tm4221)* mutation into account, these phenotypic similarities of *spin-4* and *nhr-114* mutants imply that *spin-4* and *nhr-114* function in the same pathway.

We next explored whether *spin-4* functions in the “B12-one-carbon cycle-PC” axis. We examined the level of B12 with the widely used *Pacdh-1::GFP* reporter [26, 27]. Expression of this reporter is inversely correlated with the organismal B12 level. Expression of *Pacdh-1::GFP* in wild-type animals was lower when the diet was normal HT115 compared to fresh OP50 (Supplementary Fig. S2b). The expression of *Pacdh-1::GFP* was increased in *spin-4(xd458)* mutants (Supplementary Fig. S2c and d). This result indicates that similar to *nhr-114*, *spin-4* mutants have a low organismal B12 level. Indeed, supplying B12 to fresh OP50 fully suppressed the sterility of *spin-4(xd458)* mutants (Fig. 3e). Supplementation with methionine or choline also fully suppressed the sterility of *spin-4(xd458)* and *nhr-114(gk849)* mutants (Fig. 3f and Supplementary Fig. S2e). These results demonstrate that *spin-4* functions in the “B12-one-carbon cycle-PC” axis (Fig. 3g).

We next asked how *spin-4* regulates the “B12-one-carbon cycle-PC” axis. Extracellular B12 bound with a carrier protein is firstly taken up by the cell into its lysosome, where the carrier protein is degraded; then, free B12 is exported to cytosol by lysosomal ABC transporter for further modification before it functions as a coenzyme. It was reported that deficiency in lysosomal biogenesis or acidification causes B12 deficiency in *C. elegans* [27]. We speculated that SPIN-4, the *C. elegans* ortholog of the human lysosomal transporter SPNS1, may facilitate the transport of B12 across the lysosomal. To examine the expression and protein localization of SPIN-4, we created a *Pspin-4::GFP* transcriptional fusion reporter and a *Pspin-4::spin-4::GFP* translational fusion reporter. The transcriptional GFP reporter was widely expressed, including in intestine and hypodermis (Supplementary Fig. S2f). The SPIN-4::GFP signal surrounded the signal from the lysosomal protease R07E3.1::mCherry reporter, which suggests that SPIN-4 is located on the lysosomal membrane (Supplementary Fig. S2g). Together, these results suggest that SPIN-4 likely facilitates lysosomal B12 transport and affects the “one-carbon cycle-PC” pathway.

### *nhr-114* and *spin-4* suppress the embryonic lethality of *seip-1(tm4221)* mutants through the “B12-one-carbon cycle-PC” axis

**Figure 4:**
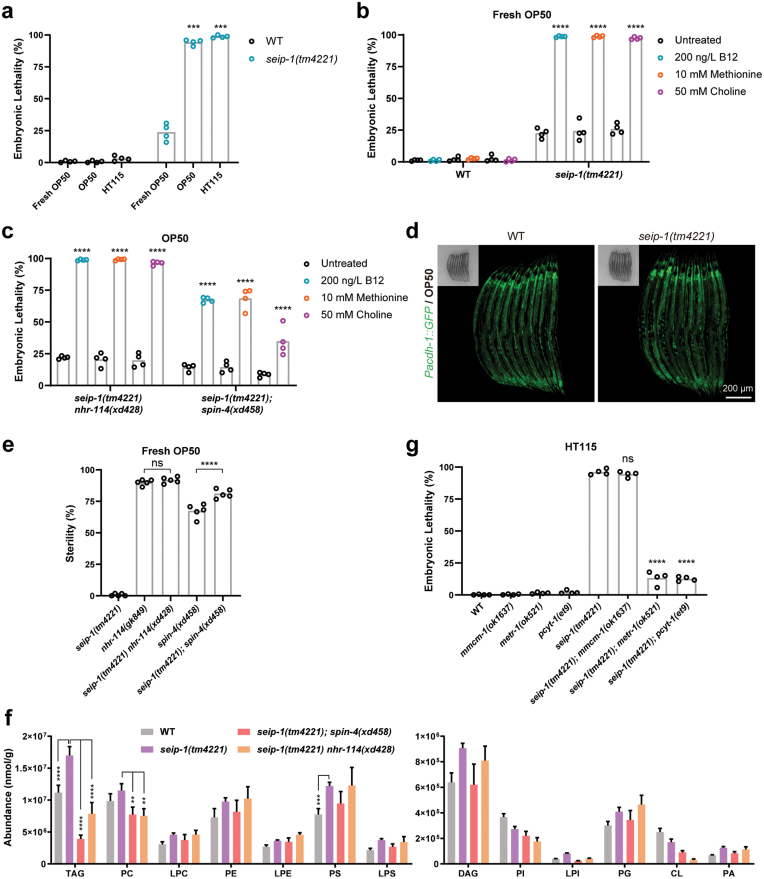
PC deficiency suppresses the embryonic lethality of the *seip-1* mutants. (a) Quantification of the embryonic lethality of wild type and *seip-1(tm4221)* mutants fed on fresh OP50, normal OP50, or normal HT115. Each point represents a biological repeat. Statistically significant differences between *seip-1(tm4221)* mutants grown on fresh OP50 and each other diet were determined by Brown-Forsythe and Welch ANOVA with *post hoc* Dunnett’s T3 test, ***P < 0.001. (b) Quantification of the embryonic lethality of wild type and *seip-1(tm4221)* mutants cultured on fresh OP50 supplied with or without 200 ng/L B12, 10 mmol/L methionine, or 50 mmol/L choline. Each point represents a biological repeat. Statistically significant differences between *seip-1(tm4221)* mutants with and without each supplemented metabolite were determined by ordinary one-way ANOVA with *post hoc* Sidak’s test, ****P < 0.0001. (c) Quantification of the embryonic lethality of *seip-1(tm4221) nhr-114(xd428)* and *seip-1(tm4221);spin-4(xd458)* double mutants cultured on fresh OP50 supplied with or without 200 ng/L B12, 10 mmol/L methionine, or 50 mmol/L choline. Each point represents a biological repeat. Statistically significant differences between mutants with and without each supplemented metabolite were determined by two-way ANOVA with *post hoc* Tukey’s test, ****P < 0.0001. (d) Confocal images of the *Pacdh-1::GFP* reporter in the wild type and *seip-1(tm4221)* background. Insets are bright-field images. (e) Sterility was compared among different mutant animals fed on the fresh OP50 diet. Each point represents a biological repeat. Statistical significance was determined by ordinary one-way ANOVA with *post hoc* Sidak’s test, ****P < 0.0001, ns, not significant. (f) Profiles of the lipid content in embryos from gravid adults of wild type, *seip-1(tm4221)*, *seip-1(tm4221) nhr-114(xd428)*, and *seip-1(tm4221);spin-4(xd458)*. Data were normalized to total protein. Error bars represent SEM. Statistically significant differences between *seip-1(tm4221)* and each other sample were determined by two-way ANOVA with *post hoc* Dunnett’s test, **P < 0.01, ***P < 0.001, and ****P < 0.0001. (g) Quantification of the embryonic lethality of wild type and mutants fed on the normal HT115 diet. Each point represents a biological repeat. Statistically significant differences between the *seip-1(tm4221)* single mutant and each double mutant were determined by ordinary one-way ANOVA with *post hoc* Dunnett’s test, ****P < 0.0001.

We then asked whether reducing the activity of the “B12-one-carbon cycle-PC” axis suppresses the embryonic lethality of *seip-1(tm4221)* mutants. Notably, fresh OP50 significantly decreased the embryonic lethal phenotype compared to normal OP50 and normal HT115 (Fig. 4a). This result indicates that the embryonic lethality of *seip-1(tm4221)* mutants is diet-dependent. Since both SPIN-4 and NHR-114 regulate the “B12-one-carbon cycle-PC” axis, we supplied *seip-1(tm4221)* mutants with B12, methionine, and choline. All of the metabolites fully blunted the alleviating effect of fresh OP50 on the embryonic lethality of *seip-1(tm4221)* mutants (Fig. 4b). Importantly, supplementation of these metabolites also significantly reduced the suppressing effect of *nhr-114(xd428)* and *spin-4(xd458)* mutations on the embryonic lethality of *seip-1(tm4221)* mutants (Fig. 4c). This suggests that the suppression effect of *nhr-114* and *spin-4* on *seip-1(tm4221)* mutants is indeed mediated through the “B12-one-carbon cycle-PC” axis.

The suppression effect of PC prompted us to examine whether the activity of the “B12-one-carbon cycle-PC” axis is increased in *seip-1(tm4221)* mutants. We examined the expression of the *Pacdh-1::GFP* reporter in *seip-1(tm4221)* mutants, and found no difference compared to the controls (Fig. 4d). This suggests that *seip-1* deficiency does not affect the organismal B12 level. We also examined whether *seip-1(tm4221)* mutation affects the sterility of *nhr-114* and *spin-4* mutants. The sterility of *nhr-114(xd428)* and *spin-4(xd458)* mutants was not suppressed by the *seip-1(tm4221)* mutation (Fig. 4e). Therefore, the activity of the “B12-one-carbon cycle-PC” axis is probably not increased in *seip-1(tm4221)* mutants.

To examine the changes in PC levels associated with suppression of the *seip-1(tm4221)* mutation, we performed lipid profiling to measure the levels of PC and other lipids in *seip-1(tm4221)* single mutant embryos and in *seip-1(tm4221) nhr-114(xd428)* and *seip-1(tm4221);spin-4(xd458)* double mutant embryos. Compared to wild type, the levels of diacylglycerol (DAG) and triacylglycerol (TAG) were dramatically increased in *seip-1(tm4221)* mutant embryos (Fig. 4f). The most abundant phospholipids, PC, PE, and PS, were also slightly increased in *seip-1(tm4221)* mutants. In *seip-1(tm4221) nhr-114(xd428)* and *seip-1(tm4221);spin-4(xd458)* double mutants, the levels of PE, PS, and other lipids were not changed, compared to *seip-1(tm4221)* alone (Fig. 4f). Consistent with the notion that suppression of the *seip-1(tm4221)* mutation occurs by reducing the activity of the “B12-one-carbon cycle-PC” axis, the level of PC was significantly decreased in both *seip-1(tm4221) nhr-114(xd428)* and *seip-1(tm4221);spin-4(xd458)* double mutants (Fig. 4f). In addition, the level of TAG was also decreased in these double mutants, compared to *seip-1(tm4221)* alone. In line, the mutation *metr-1(ok521)*, which affects the one-carbon cycle, and the mutation *pcyt-1(et9)*, which affects PC synthesis, significantly suppressed the embryonic lethality of *seip-1(tm4221)* mutants, while the mutation *mmcm-1(ok1637)*, which affects the propionate breakdown pathway, did not (Fig. 4g). Put together, these results support the idea that lowering the activity of the “B12-one-carbon cycle-PC” axis suppresses the embryonic lethality of *seip-1(tm4221)* mutants.

### Suppression of the embryonic lethality of *seip-1* mutants by PC deficiency depends on PUFAs

**Figure 5:**
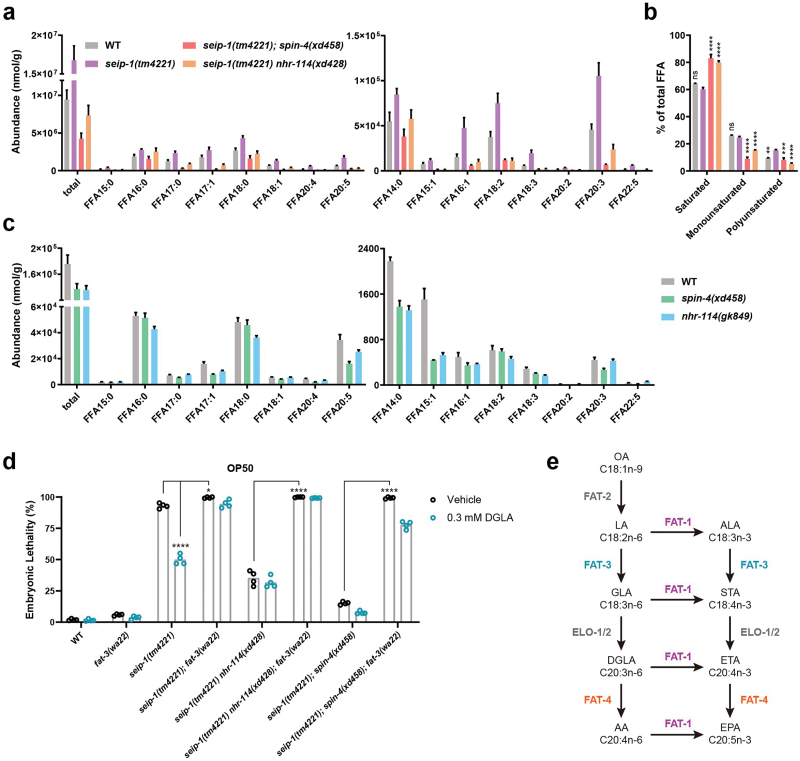
Suppression of the embryonic lethality of the *seip-1* mutants by PC deficiency depends on PUFAs. (a) Profiles of FFAs in early embryos of wild type, *seip-1(tm4221)*, *seip-1(tm4221) nhr-114(xd428)*, and *seip-1(tm4221);spin-4(xd458)*. Data were normalized to total protein. Error bars represent SEM. (b) Relative levels to total FFAs of saturated FFAs, monounsaturated FFAs, and polyunsaturated FFAs in embryos from gravid adults of wild type, *seip-1(tm4221)*, *seip-1(tm4221) nhr-114(xd428)*, and *seip-1(tm4221);spin-4(xd458)*. Error bars represent SEM. Statistically significant differences between *seip-1(tm4221)* and each other sample were determined by two-way ANOVA with *post hoc* Dunnett’s test, **P < 0.01, ***P < 0.001, and ****P < 0.0001. (c) Profiles of FFAs in day 1 adults of wild type, *spin-4(xd458)* mutants and *nhr-114(gk849)* mutants. Data were normalized to total protein. Error bars represent SEM. (d) Quantification of the embryonic lethality of wild type and mutants fed on the OP50 diet supplied with 0.3 mmol/L DGLA or vehicle. Each point represents a biological repeat. Statistical significances were determined by two-way ANOVA with *post hoc* Tukey’s test, *P < 0.05 and ****P < 0.0001. (e) The pathway for fatty acid elongation and desaturation in *C. elegans*. Enzymes that catalyze each step are indicated alongside the arrows. POA, palmitoleic acid; VA, vaccenic acid; PA, palmitic acid; SA, stearic acid; OA, oleic acid; LA, linoleic acid; ALA, alpha-linolenic acid; GLA, gamma-linoleic acid; STA, stearidonic acid; DGLA, dihomogamma-linolenic acid; ETA, eicosatetraenoic acid; AA, arachidonic acid; EPA, eicosapentaenoic acid.

We next explored the underlying mechanism of the suppression of PC deficiency on *seip-1(tm4221)* embryonic lethality. Recent studies reported that dietary supplementation of GLA(C18:3n-6) and DGLA(C20:3n-6), two ω-6 PUFAs, promote the enrichment of SEIP-1 in an ER subdomain and partially rescues the embryonic lethality of *seip-1* mutants [18, 28]. To investigate whether *nhr-114* and *spin-4* mutations suppressed the embryonic lethality of *seip-1(tm4221)* mutants by increasing the level of PUFAs, we analyzed the abundance of free fatty acids (FFAs). The levels of FFAs were increased in *seip-1(tm4221)* embryos and decreased in *seip-1(tm4221) nhr-114(xd428)* and *seip-1(tm4221);spin-4(xd458)* double mutant embryos (Fig. 5a). Among FFAs, we also compared the relative levels of saturated fatty acids (SFAs), monounsaturated fatty acids (MUFAs) and PUFAs. While SFA and MUFA levels were not changed in *seip-1(tm4221)* mutants compared to wild type, PUFA levels were increased in *seip-1(tm4221)* embryos. In *seip-1(tm4221) nhr-114(xd428)* and *seip-1(tm4221);spin-4(xd458)* double mutant embryos, SFA levels were increased, and MUFA and PUFA levels were decreased compared to *seip-1(tm4221)* alone (Fig. 5b). The abundance of C20:3 fatty acids were dramatically increased in *seip-1(tm4221)* mutants. In both double mutant embryos, the abundance of C20:3 fatty acids were dramatically decreased compared to *seip-1(tm4221)* single mutants (Fig. 5a). In addition, the abundance of most FFAs was unchanged or even decreased in *nhr-114(gk849)* and *spin-4(xd458)* single mutants (Fig. 5c). These data suggest that the suppression effect of *nhr-114* and *spin-4* mutations on the embryonic lethality of *seip-1(tm4221)* is unlikely to occur through increasing the levels of DGLA or other PUFAs.

We further examined the relationship between *nhr-114*/*spin-4*-mediated suppression and PUFAs. Consistent with a previous report [18], DGLA supplementation partially suppressed the embryonic lethality of *seip-1(tm4221)* mutants (Fig. 5d). Interestingly, DGLA supplementation did not enhance the suppression effect of the *nhr-114(xd428)* on *seip-1(tm4221)* mutations. The suppression effect of the *spin-4(xd458)* mutation on *seip-1(tm4221)* mutants was slightly enhanced by DGLA supplementation (Fig. 5d). *De novo* PUFA biosynthesis starts from the desaturation of oleic acid by FAT-2, followed by three other desaturases, FAT-1, FAT-3 and FAT-4, to generate complex PUFAs [29] (Fig. 5e). The *fat-3(wa22)* mutation, which blocks the synthesis of downstream PUFAs including DGLA, enhanced the embryonic lethality of *seip-1(tm4221)* mutants and blocked the rescue effect of DGLA supplementation (Fig. 5d). Notably, the *fat-3(wa22)* mutation completely abolished the suppression effect of *nhr-114* and *spin-4* mutations on the embryonic lethality of *seip-1(tm4221)* mutants (Fig. 5d). Together, these results indicate that the rescuing effect of PC deficiency on the embryonic lethality of *seip-1(tm4221)* mutants depends on PUFAs.

### The embryonic lethality of *seip-1* mutants is regulated by membrane lipid homeostasis

**Figure 6:**
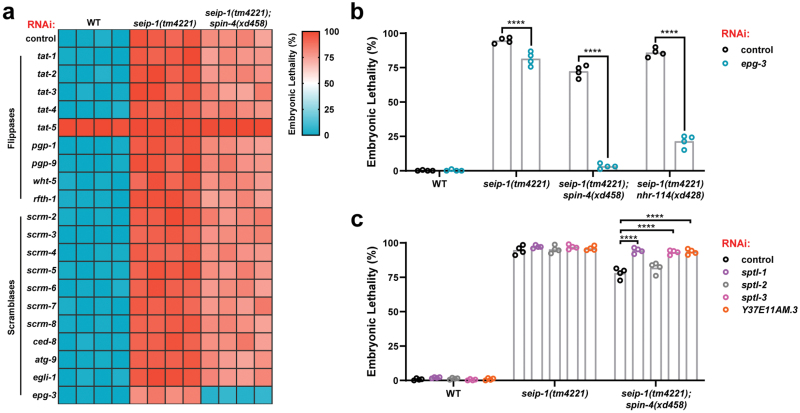
The embryonic lethality of the *seip-1* mutants is regulated by membrane lipid homeostasis. (a) Heatmap of the embryonic lethality of wild type and mutants fed on the different RNAi bacteria targeting the putative flippases and scramblases. Each tile represents a biological repeat. (b) Quantification of the embryonic lethality of wild type and mutants fed on different RNAi bacteria. Each point represents a biological repeat. Statistical significances were determined by two-way ANOVA with *post hoc* Sidak’s test, ****P < 0.0001. (c) Quantification of the embryonic lethality of wild type and mutants fed on different RNAi bacteria targeting the enzymes for the *de novo* biosynthesis of sphingolipids. Each point represents a biological repeat. Statistical significances were determined by two-way ANOVA with *post hoc* Dunnett’s test, ****P < 0.0001.

We speculated that PC and PUFAs might regulate the embryonic lethality of *seip-1(tm4221)* mutants by affecting the homeostasis of membrane lipids. We examined the genetic interaction between *seip-1* and a panel of flippases and scramblases, which shapes the membrane phospholipid bilayer. A set of *C. elegans* homologs of human flippases and scramblases was screened by RNAi knockdown. Except *tat-5* and *epg-3*, most knockdowns did not affect the wild type and *seip-1(tm4221);spin-4(xd458)* animals (Fig. 6a). *tat-5* knockdown caused embryonic lethal in wild type and *seip-1(tm4221)* mutants. Interestingly, knockdown of *epg-3*, which encodes a homolog of human VMP1, ameliorated the embryonic lethality of *seip-1(tm4221)* and *seip-1(tm4221);spin-4(xd458)* double mutants (Fig. 6a). The suppression effect of *epg-3* knockdown was confirmed by independent experiments on *seip-1(tm4221)*, *seip-1(tm4221);spin-4(xd458)*, and *seip-1(tm4221) nhr-114(xd428)* mutants (Fig. 6b). Notably, *epg-3* depletion completely suppressed the embryonic lethality of *seip-1(tm4221);spin-4(xd458)* animals (Fig. 6b). EPG-3/VMP1 is an ER-resident phospholipid scramblase and regulates ER membrane dynamics [30–32]. Taken together, these results suggest that ER phospholipid homeostasis is important for the embryonic development of *seip-1(tm4221)* mutants.

Previous studies in yeast revealed that seipin inhibits the biosynthesis of sphingolipids [33], which provoked us to investigate the role of sphingolipids in the embryogenesis of *seip-1(tm4221)* mutants. Serine palmitoyltransferase (encoded by *sptl-1*, *sptl-2*, and *sptl-3*) and 3-ketodihydrosphingosine reductase (encoded by *Y37E11AM.3*) were knocked down to inhibit the synthesis of long-chain base, which is the basic structure of sphingolipids. Blockage of sphingolipid biogenesis did not alleviate the embryonic lethality of *seip-1(tm4221)* mutants but rather repressed the suppressive effect of *spin-4(xd458)* mutation on that of *seip-1(tm4221)* mutants (Fig. 6c). Therefore, it is unlikely that SEIP-1 deficiency induces embryonic lethality through increased sphingolipid levels. Instead, sphingolipids may regulate the embryogenesis of *seip-1(tm4221)* mutants through affecting membrane lipid homeostasis.

### PC deficiency enhances the lipid droplet phenotype of *seip-1* mutants

**Figure 7:**
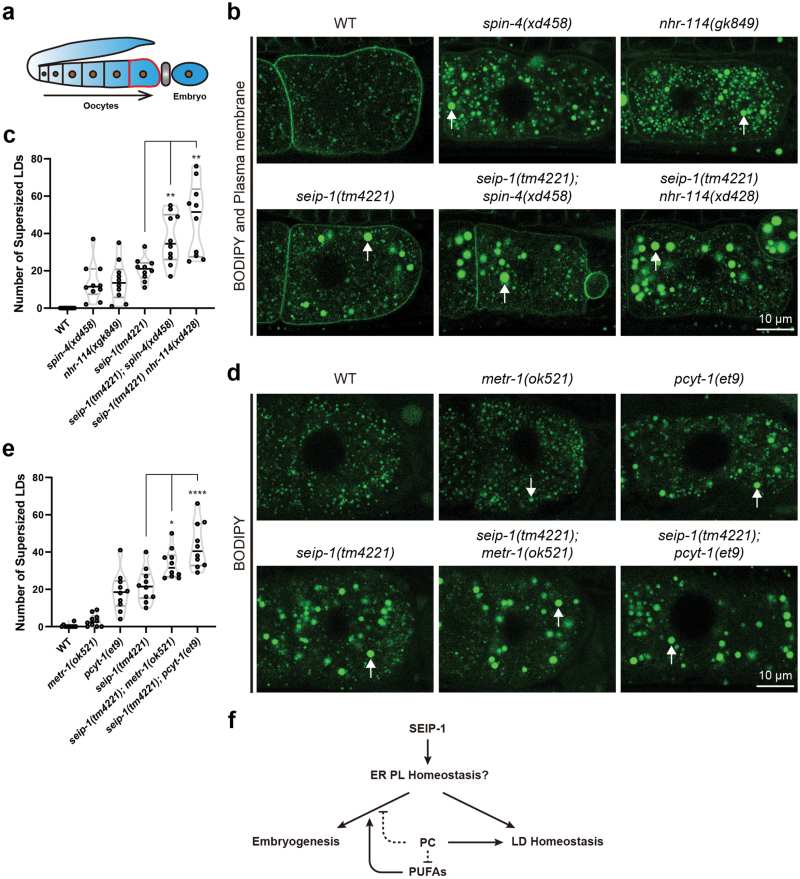
PC deficiency enhances the lipid droplet phenotype of the *seip-1* mutants. (a) Diagram of the development of *C. elegans* oocytes. The plasma membrane of a mature oocyte that is ready for fertilization is displayed as red. The spermatheca is indicated in gray. (b) Representative confocal images of supersized lipid droplets in mature oocytes of wild type and mutants. White arrows indicate supersized lipid droplets. (c) Quantification of the supersized lipid droplets in B. Each point represents a biological repeat. Statistically significant differences between the *seip-1(tm4221)* single mutant and each double mutant were determined by Brown-Forsythe and Welch ANOVA with *post hoc* Dunnett’s T3 test, **P < 0.01. (d) Representative confocal images of supersized lipid droplets in mature oocytes of wild type and the mutants defective in PC synthesis. White arrows indicate supersized lipid droplets. (e) Quantification of the supersized lipid droplets in D. Each point represents a biological repeat. Statistically significant differences between the *seip-1(tm4221)* single mutant and each double mutant were determined by ordinary one-way ANOVA with *post hoc* Dunnett’s test, *P < 0.05 and ****P < 0.0001. (f) Working model for the role of PC, PUFAs and SEIP-1 in the regulation of embryogenesis and lipid droplet homeostasis. ER, endoplasmic reticulum; PL, phospholipid; PC, phosphatidylcholine; PUFA, polyunsaturated fatty acid; LD, lipid droplet.

The suppression effect of *spin-4(xd458)* and *nhr-114(xd428)* mutations on the embryonic lethality of *seip-1(tm4221)* mutants provides a great opportunity to address a longstanding question about seipin: what is the relationship between its roles in cellular lipid droplet homeostasis and physiological function (i.e. embryogenesis in this study). In mature oocytes (Fig. 7a), *seip-1(tm4221)* mutants exhibit numerous large lipid droplets compared to wild type (Fig. 7b and c). This “supersized” lipid droplet phenotype is also found in yeast, fly and mouse *seipin* mutants [3, 4, 20, 34–36].

We then examined the effect of *spin-4(xd458)* and *nhr-114(xd428)* mutations on lipid droplets in *seip-1(tm4221)* mutant oocytes. Similar to *seip-1(tm4221)* mutants, *spin-4(xd458)* and *nhr-114(xd428)* mutants exhibited large lipid droplets (Fig. 7b and c), consistent with previous findings that PC deficiency leads to large lipid droplets [12, 37, 38]. Remarkably, *spin-4(xd458)* and *nhr-114(xd428)* mutations further increased the number of large lipid droplets in *seip-1(tm4221)* oocytes (Fig. 7b and c). In line with that, the mutants *metr-1(ok521)* and *pcyt-1(et9)*, which are defective in PC synthesis, also displayed large lipid droplets. The number of large lipid droplets was also significantly increased in *seip-1(tm4221);metr-1(ok521)* and *seip-1(tm4221);pcyt-1(et9)* double mutants compared to *seip-1(tm4221)* single mutants (Fig. 7d and e). Therefore, while PC deficiency suppresses the embryonic lethality in *seip-1(tm4221)* mutants, it exacerbates the large lipid droplet phenotype in *seip-1(tm4221)* mutants. These results suggest that seipin may regulate embryogenesis and lipid droplet biogenesis through distinct mechanisms (Fig. 7f).

## Discussion

In *seipin*-deficient organisms, the causal link between the cellular defect in lipid droplet homeostasis and the physiological defects is not clear. This study reveals that *seip-1* embryonic lethality is suppressed by reducing PC synthesis. *nhr-114*, a known regulator of the “B12-one carbon cycle-PC” axis, and *spin-4*, a new player in that axis, were identified as *seip-1* suppressors. The choline-phosphate cytidylyltransferase mutant *pcyt-1*, which is defective in PC synthesis, also suppresses the embryonic lethality of *seip-1* mutants. Consistent with previous reports [12, 34, 35], both *seip-1* mutation and PC deficiency mutations result in large lipid droplets. Interestingly, while PC deficiency suppresses *seip-1* embryonic lethality, it enhances the large lipid droplet phenotype of *seip-1* mutants. Therefore, our results suggest that seipin-mediated embryogenesis is independent of lipid droplet homeostasis (Fig. 7f).

The suppression of *seip-1* embryonic lethality by reduction of PC is unexpected. PC is a major phospholipid on lipid droplets. While reducing PC level results in large lipid droplets from lipid droplet coalescence [12], the formation of lipid droplets is also an adaptation to PC deficiency [28, 37, 39, 40]. The *seipin* mutation also results in aberrant lipid droplet biogenesis with the formation of large lipid droplets, a phenotype similar to PC deficiency. Therefore, it is quite surprising that reducing PC synthesis suppresses the physiological defect of *seip-1* mutants. Similar to PC, the link between PUFA and lipid droplet dynamics is also obvious [41, 42]. Notably, PUFA affects lipid droplet diversity by controlling the localization of seipin [28]. Although the PUFA composition is changed in mouse and worm *seipin* mutants [18, 28, 43], this cannot explain the suppression of the *seip-1* mutant phenotype by PUFA supplementation. Besides, inhibiting PUFA synthesis represses lipid droplet (LD) defects induced by hepatic seipin deficiency [44]. Together, PUFA is likely to have opposite actions on the molecular and physiological defects induced by seipin deficiency. The suppression of *seip-1* embryonic lethality by PC reduction requires PUFA. The mechanistic link between PC and PUFA is not clear here. It is possible that PC and PUFA act in parallel, but converge on the same target/process. Alternatively, PC may directly inhibit PUFA function (Fig. 7f).

We propose two possibilities to reconcile the lipid droplet homeostasis function and embryogenesis function of seipin. First, seipin may be multifunctional. Besides its role in lipid droplet biogenesis, seipin may function in other processes. In line with that, yeast seipin inhibits sphingolipid biogenesis. This function is parallel to its role in lipid droplet biogenesis, because inhibition of sphingolipid biogenesis only has a minor effect on lipid droplet biogenesis [33]. The diverse functions of seipin may be mediated by its binding with different partners [33, 34, 45, 46]. Another possibility is that seipin acts in a step before lipid droplet homeostasis, and this step is shared with both lipid droplet homeostasis and embryogenesis/lipid barrier synthesis (Fig. 7f). Consistent with this hypothesis, biogenesis of lipid droplets that contain only retinyl or steryl esters does not depend on seipin [47], which suggests that seipin does not directly determine droplet formation. It is possible that seipin regulates ER phospholipid homeostasis to counteract local phospholipid imbalances, membrane curvature changes, or thermodynamic instability of neutral lipids in lipid bilayers to facilitate lipid droplet formation and lipid barrier synthesis/formation in an ordered and regulated way.

Seipin may affect ER membrane lipid homeostasis directly by acting as a phospholipid synthetase/lipid transporter/flippase/scramblase or as a modulator of these functions [13, 30–32]. For example, seipin binds to GPAT3/4 (glycerol-3-phosphate acyltransferase) and negatively modulates its activity. Seipin deficiency leads to increased levels of phosphatidic acid, a conical membrane phospholipid, which promotes the negative curvature of membranes [34–36]. Genetic evidences in this study and others are in line with this possibility. First, EPG-3/VMP1 deficiency suppresses the embryonic lethality of *seip-1* mutants. EPG-3/VMP1 is a resident protein in ER membrane that functions as a phospholipid scramblase [30, 32]. In addition, both seipin and EPG-3/VMP1 were identified as partners of SERCA [31, 48]. Seipin and VMP1 may regulate phospholipid homeostasis at the same ER subdomain. Second, PC reduction or PUFA supplementation also suppresses seipin deficiency. PC is a membrane-forming cylindrical phospholipid, and therefore reducing PC affects membrane packing, permeability, and intrinsic membrane curvature, as well as inducing acyl chain remodeling of the remaining phospholipids [49–53]. Long-chain PUFAs are conformationally flexible, and their incorporation into phospholipids greatly changes the physicochemical properties of membranes. Finally, blocking the synthesis of sphingolipids represses the suppression of PC reduction on the embryonic lethality of *seip-1* mutants. Given that sphingolipids are mainly synthesized at ER membrane and regulated by seipin [33], it is possible that their contents influence ER membrane homeostasis.

Alternatively, seipin may affect ER phospholipid homeostasis indirectly, such as through ER-lipid droplet contacts, which may maintain ER membrane homeostasis by removal of excessive lipids from ER membrane or providing lipids from the lipid droplet to the ER [41]. A recent study suggested that Rab18 rescues the embryonic lethality of *seip-1* mutants by targeting to the lipid droplets [54]. The specific localization of Rab18 promotes the contact between lipid droplets and the ER [55, 56]. Interestingly, PC reduction, PUFA supplementation, and VMP1 deficiency may also increase ER-lipid droplet contacts [28, 31].

The suppression of the embryonic lethality is likely due to recovery of the lipid barrier in the eggshell [21, 22]. It is possible that seipin-regulated ER membrane lipid balance is required for lipid barrier construction (the synthesis of specific lipids or the formation of the barrier). VMP1 deficiency, PC reduction, and PUFA supplementation may remodel the membrane lipid composition and change membrane properties to facilitate eggshell lipid barrier construction. Interestingly, VMP1 was recently reported to regulate the secretion of lipoprotein particles, which resemble lipid droplets structurally and are also assembled in the ER membrane but are released into the ER lumen for secretion [57].

There have been several attempts to restore the normal physiological behaviors of seipin-deficient mice. These studies either directly target different physiological defects associated with seipin deficiency or originate from suppression of the lipid droplet phenotype [58–60]. Our finding raises the possibility that the well-studied cellular lipid droplet defect and physiological defects caused by seipin deficiency may be separable. The autonomous action of seipin supports this notion [61, 62]. An immediate application is to treat seipin deficiency with an inhibitor of PC synthesis. In addition, dietary manipulation of the B12, methionine or choline level is another appealing intervention for physiological defects in BSCL2 patients. The results may be beneficial for future treatment of diseases associated with *seipin* mutations and may extend to other diseases.

## Materials and methods

### Strains and maintenance

The *C. elegans* strains used in this study are listed in Supplementary Table S1. Bristol N2 was used as wild-type control. All *C. elegans* strains were cultured at 22°C on nematode growth medium (NGM) plates. *E. coli* OP50 was seeded onto fresh NGM plates for 2 days to allow lawn formation, and this was defined as the “fresh OP50” diet. *E. coli* OP50 and *E. coli* HT115 were seeded onto fresh NGM plates for 7 days, and these were defined as the “normal OP50 or HT115” diets. The diets can be stored at 4°C for no more than 1 week before utilization.

### EMS screen and suppressor identification

For the suppressor screen, P0 L4 larvae of *seip-1(tm4221)/nT1[qIs51]* mutants were mutagenized by EMS. Every 3 F1 larvae were transferred to a new OP50 plate, and every 10 F2 larvae that no longer contained balancer fluorescence were cultured on a new OP50 plate. F2 plates that contained obviously increased numbers of F3 larvae were kept. The putative suppressors from F2 plates were transferred to new OP50 plates and were selected for increased progeny numbers through several generations.

To map the chromosomal location of the suppressors, the suppressors were crossed with the CB4856 strain, and each F2 animal was cultured alone on an OP50 plate. F2 animals that were homozygous for the *seip-1(tm4221)* mutation were classified into two groups according to their progeny number. Then single nucleotide polymorphism mapping was applied to these two groups as described [63].

The genomic DNA of suppressor-containing strains was extracted for whole-genome sequencing. The candidate suppressor genes were selected according to the mapping and sequencing results. To knock out *nhr-114* via CRISPR-Cas9 technology, two sgRNA sequences targeting the first exon of *nhr-114* were designed and validated for specificity through BLAST searching against the whole genome. The sgRNA sequences were inserted into pDD162 plasmid and sequenced for validity. Due to the close linkage of *nhr-114* and *seip-1*, the recombinant plasmids were microinjected into *seip-1(tm4221)* mutant animals. Offspring were singled to examine the mutation status by sequencing. The homozygous mutant, *seip-1(tm4221) nhr-114(xd428)*, was obtained in next generation along with the loss of the Cas9 plasmid. A point mutation in *spin-4(xd458)* was generated from wild-type animals through CRISPR-Cas9 by SunyBiotech company. Then the *spin-4(xd458)* mutation was crossed into *seip-1(tm4221)* mutant to generate *seip-1(tm4221);spin-4(xd458)* double mutants.

### Molecular biology

The genomic DNA of *seip-1* was amplified from N2 genomic DNA. The forward primer was 5’-catcacgtgttcgctcgctgg-3’ and the reverse primer was 5’-tgccgacgaggacggttcgac-3’. The transgenic worm *seip-1(tm4221);xdEx1640(Pseip-1::seip-1 + rol-6[su1006])* was generated by microinjecting the amplified *seip-1* genomic DNA with a coinjection marker. Approximately 3.5 kb of upstream sequence of *spin-4* was cloned from wild-type genomic DNA and inserted into the multiple cloning site of pSM plasmid to generate the *spin-4* promoter fusion GFP reporter *Pspin-4::GFP*. The reporter was expressed in wild-type animals by microinjection with a coinjection marker to generate *xdEx2409 [Pspin-4::GFP + Podr-1::RFP]* animals. The coding sequence of SPIN-4 isoform a was cloned from wild-type cDNA and inserted in-frame between the *vha-6* promoter and GFP-coding sequence of pSM::*Pvha-6::GFP* through *in vitro* recombination to generate the *Pvha-6*::*spin-4::GFP* reporter. The reporter was expressed in *qxIs448 [R07E3.1::mCherry]* animals that express a mCherry-labeled lysosomal peptidase by microinjection with a coinjection marker to generate *qxIs448 [R07E3.1::mCherry];xdEx2483 [Pvha-6::SPIN-4::GFP + Podr-1::RFP]* animals.

### Quantification of physiological phenotypes

To quantify embryonic lethality, synchronized L1 larvae were cultured to day 1 adults (24 hours post mid-L4 stage). For each genotype or treatment, four independent plates were cultured. Twenty to thirty adult animals per plate were picked to a new plate and allowed to lay embryos for 2 − 3 hours. Then the animals were removed, and the total number of embryos was counted. After 20 hours, the dead embryos were counted. Embryonic lethality was calculated as the percentage of dead embryos among total embryos. To determine brood size, a single L4 animal was transferred to a new OP50 plate. Every 24 hours since the start of adulthood, the animal was transferred to a new plate, and the embryos and larvae on the previous plate were counted. For each genotype, 10 − 20 L4 animals were singled for the analysis. To quantify sterility, synchronized L1 larvae were cultured to day 1 adults. For each genotype or treatment, five independent plates were cultured. More than 50 adults were picked per plate for inspection of *in utero* embryos under differential interference contrast microscopy (DIC). Sterile animals were characterized by an empty uterus. Sterility was calculated as the percentage of sterile animals among total animals.

Hypertonic solution (250 mmol/L KCl, 5 mmol/L HEPES) or hypotonic solution (100 mmol/L KCl, 5 mmol/L HEPES) was used to identify the integrity of the eggshell. Embryos were released from gravid adults by cutting the animals with two 1 mL syringe needles in the tested solution within an artificial hole in a 2.5% agarose pad to relieve the pressure from coverslip. Representative DIC images of two-cell embryos were captured by ZEISS microscopy. Eggshell permeability was quantified by calculating the percentage of shrunken embryos among total embryos in hypertonic solution. For each genotype, four repeats were performed, each with 100 embryos in total.

### Staining and imaging

For DAPI staining, embryos were directly released from gravid adults in a droplet of DAPI (ThermoFisher Scientific) solution (2 μg/mL in M9 buffer) on a microscope slide. The slides were kept in a wet box and stained for 20 min in the dark. To visualize lipid droplets in embryos, the embryos released from gravid adults were stained in BODIPY 493/503 (ThermoFisher Scientific) solution (5 μg/mL in M9 buffer) for 20 min. After one wash with M9 buffer, stained embryos were transferred onto a 2.5% agarose pad and sealed by a coverslip. The samples were immediately imaged by fluorescence microscopy or confocal microscopy. To visualize yolk particles in embryos, the embryos were immediately imaged by confocal microscopy after being released from gravid adults.

To visualize lipid droplets in oocytes, synchronized day 1 adults were washed off from culture plates with M9 buffer. The supernatant was discarded, then the animals were resuspended in BODIPY solution and stained in the dark for 3 hours with gentle shaking. The animals were then transferred to a new food plate to recover for 3 hours. The lipid droplets in mature oocytes were imaged by confocal microscopy. The numbers of supersized lipid droplets (diameter > 1 μm) were counted over a 14-μm z-axis thickness per mature oocyte. Ten oocytes were analyzed for each genotype.

To image embryos labeled by reporters for the plasma membrane and nucleus, embryos were released from gravid adult animals in a drop of M9 buffer. The embryos were transferred onto a 2.5% agarose pad, and a coverslip was lightly placed over them. Next, the edge of the coverslip was sealed by wax, and the gaps around the agarose pad were filled with egg salt buffer. Then the embryos were imaged by confocal microscopy (Leica SP8).

To visualize the expression of genes in adults, synchronized L1 larvae expressing the *Pacdh-1::GFP* reporter were cultured to day 1 adults. For each genotype, 10 adult animals were picked out for confocal microscopy at a 2.5 μm z-axis interval. The final images were created by merging all the z-axis photos covering the whole animals. To quantify the expression of the *Pacdh-1::GFP* reporter in wild type and *spin-4(xd458)* mutants, three parts of the images were segmented to measure the mean intensity by ImageJ software.

To analyze the subcellular location of SPIN-4, the intestine of adult *qxIs448 [R07E3.1::mCherry];xdEx2483 [Pvha-6::SPIN-4::GFP + Podr-1::RFP]* animals were imaged by confocal microscopy. To highlight the relative location of SPIN-4 and R07E3.1, line profiles of the intensity of both GFP and mCherry were analyzed by ImageJ software.

### Electron microscopy

Day 1 adult animals were collected for high-pressure freezing EM imaging as described [64]. Sixty nanometer ultrathin sections were prepared and imaged at 80 kV using a JEM-1400 TEM (Hitachi HT7700) with a Gatan832 4k × 2.7k CCD camera.

### RNAi feeding treatment

The RNAi feeding treatment was carried out as described [65]. The synchronized L1 larvae were seeded onto RNAi plates and cultured to day 1 adults to determine the embryonic lethality. For *sptl-1* RNAi, the synchronized L1 larvae were primed by control bacteria for 24 hours to avoid developmental arrest.

### Metabolite supplementation

B12 (Sigma–Aldrich) was dissolved and diluted in ddH_2_O to make a 200 μg/L stock solution. NGM medium containing 200 ng/L B12 was made by adding 1 mL stock solution into 1 L sterile NGM before pouring plates. NGM medium containing 10 mmol/L methionine or 50 mmol/L choline was made by directly adding 1.5 g L-methionine (BioDee Biotechnology) or 7 g choline chloride (Sigma–Aldrich) respectively into 1 L sterile NGM before pouring plates. NGM medium containing 300 μmol/L DGLA (Cayman Chemicals) or 1:1000 ethanol (as control) was made by adding 1 mL DGLA stock (300 mmol/L in ethanol) or 1 mL ethanol respectively into 1 L sterile NGM before pouring plates. NGM plates containing DGLA were stored in the dark.

### Lipid profiling analysis

Animal collection: About 20,000 synchronized day 1 adults were washed off from culture plates by M9 buffer. The animals were suspended in M9 buffer to digest the food for 1 hour and then washed another three times with M9 buffer. The animal pellets were stored at −80°C until lipid analysis. Embryo collection: About 50,000 synchronized day 1 adults were washed off from culture plates by M9 buffer. The animals were lysed by bleach buffer (1.25 mol/L NaOH, 25% v/v bleach) to release the embryos. Then the embryo pellets were washed three times before being stored at −80°C until lipid analysis.

Lipids were extracted using a modified version of the Bligh and Dyer’s method as described previously [66]. Briefly, tissues were homogenized in 750 µL of chloroform:methanol 1:2 (v/v) with 10% deionized water on a Bead Ruptor (Omni, USA). The homogenate was then incubated at 1500 rpm for 1 hour at 4°C. At the end of the incubation, 350 µL of deionized water and 250 µL of chloroform were added to induce phase separation. The samples were then centrifuged, and the lower organic phase containing lipids was extracted into a clean tube. Lipid extraction was repeated once by adding 500 µL of chloroform to the remaining tissues in aqueous phase, and the lipid extracts were pooled into a single tube and dried in the SpeedVac under OH mode. Samples were stored at −80°C until further analysis.

Polar lipids were analyzed using an Agilent 1260 high performance liquid chromatography (HPLC) system coupled with a triple quadrupole/ion trap mass spectrometer (5500 Qtrap; SCIEX) [67]. Separation of individual lipid classes of polar lipids by normal phase (NP)-HPLC was carried out using a Phenomenex Luna 3 µm-silica column (internal diameter 150 × 2.0 mm) with the following conditions: mobile phase A (chloroform:methanol:ammonium hydroxide 89.5:10:0.5) and mobile phase B (chloroform:methanol:ammonium hydroxide:water 55:39:0.5:5.5). MRM transitions were set up for comparative analysis of various polar lipids. Individual lipid species were quantified by referencing to spiked internal standards, including d_31_-PC(16:0/18:1), d_31_-PE(16:0/18:1), d_31_-PG(16:0/18:1), d_31_-PS(16:0/18:1), d_7_-PI(15:0/18:1), PA 17:0/17:0, CL 80:4, LPC-d_4_-26:0, LPE 17:1, LPI 17:1, and LPS 17:1 from Avanti Polar Lipids. FFAs were quantitated using d_31_-16:0 (Sigma–Aldrich) and d_8_-20:4 (Cayman Chemicals) as internal standards. Glycerol lipids, including DAG and TAG were quantified using a modified version of reverse phase HPLC/MRM. Separation of neutral lipids was achieved on a Phenomenex Kinetex-C18 2.6 µm column (i.d. 4.6 × 100 mm) using an isocratic mobile phase containing chloroform:methanol:0.1 M ammonium acetate 100:100:4 (v/v/v) at a flow rate of 300 µL for 10 min. Levels of short-, medium-, and long-chain TAGs were calculated by referencing to spiked internal standards of TAG(14:0)_3_-d_5_, TAG(16:0)_3_-d_5_, and TAG(18:0)_3_-d_5_ obtained from CDN isotopes. DAGs were quantified using d_5_-DAG16:0/16:0 and d_5_-DAG18:1/18:1 as internal standards (Avanti Polar Lipids). To facilitate comparison between samples, the lipid content was normalized to total protein in each sample.

### Statistical analysis

All statistical analysis was done in GraphPad Prism 8.0 (GraphPad Software, Inc.). Each point stands for one independent experimental repeat and all error bars indicate SEM. Data were first inspected for normality. Nonparametric tests were applied when the data deviated from normal distribution. Otherwise, the two-tailed unpaired *t*-test was used for comparison between two groups; the test was with Welch’s correction if the groups had unequal SD. Ordinary one-way analysis of variance (ANOVA) was used for comparison among multiple groups with equal SD, and Brown-Forsythe and Welch ANOVA was used for multiple groups with unequal SD followed by an appropriate *post hoc* test for multiple comparisons (for each experiment, the tests used are stated in the figure legend). Statistically significant differences between groups with two variations were analyzed by two-way ANOVA. Statistical significances are indicated as: *P < 0.05, **P < 0.01, ***P < 0.001, ****P < 0.0001.

## Supplementary Material

loac021_suppl_Supplementary_Material

## Data and materials availability

All data needed to evaluate the conclusions in the article are present in the article and/or the Supplementary Materials. Additional data as well as materials used in this study may be requested from authors.
