# Supplementary material for: Reduced phosphatidylcholine synthesis suppresses the embryonic lethality of seipin deficiency
Source: Life Metab. 2022 Sep 8;1(2):175–89. doi: 10.1093/lifemeta/loac021 (PMC11749705; doi:10.1093/lifemeta/loac021)
Supplement: loac021_suppl_Supplementary_Material [file loac021_suppl_Supplementary_Material.pdf]

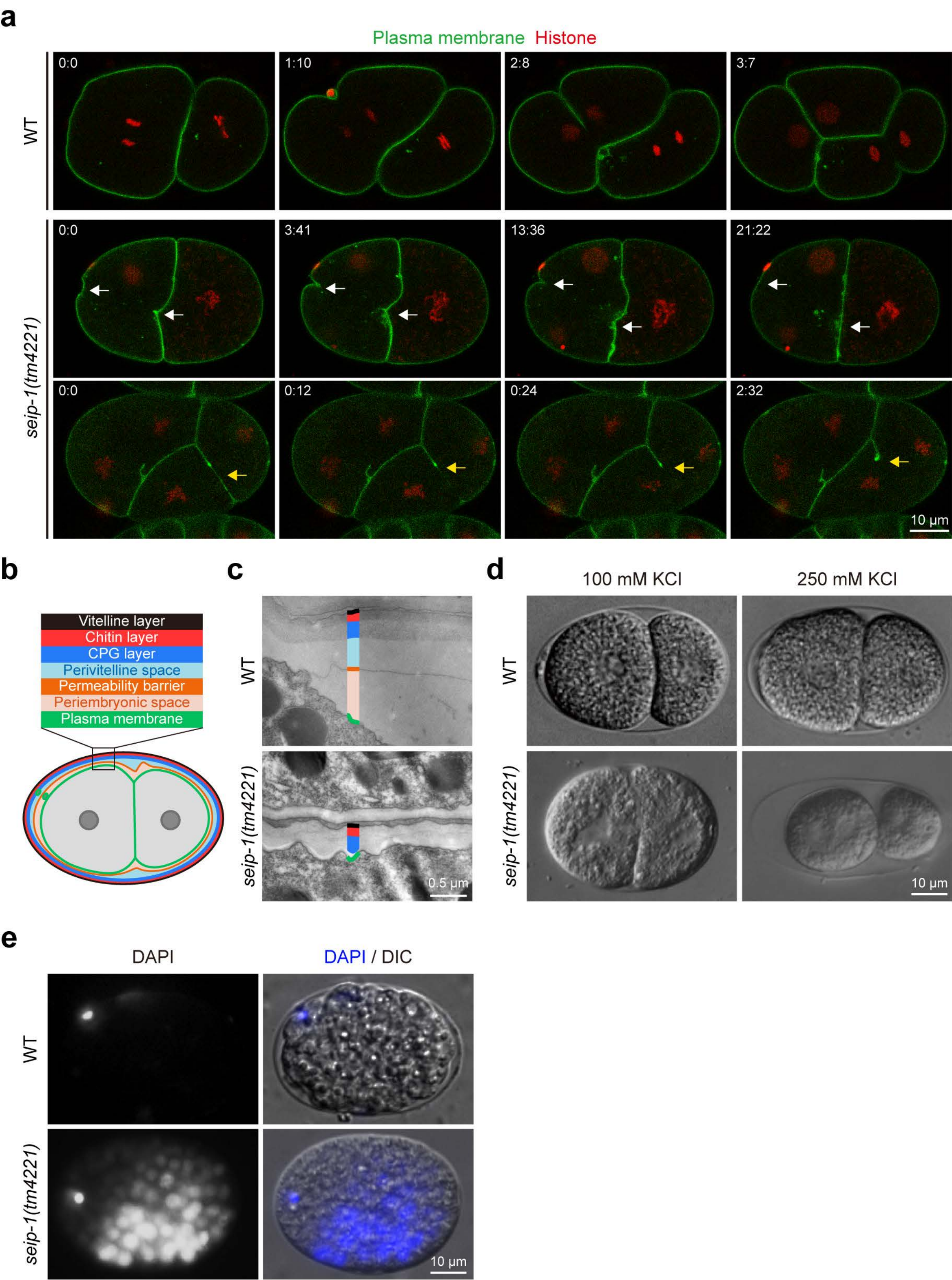
a
Plasma membrane Histone
WT
0:0
1:10
2:8
3:7
seip-1(tm4221)
0:0
3:41
13:36
21:22
0:0
0:12
0:24
2:32
10 µm
b
Vitelline layer
Chitin layer
CPG layer
Perivitelline space
Permeability barrier
Periembryonic space
Plasma membrane
c
WT
seip-1(tm4221)
0.5 µm
d
100 mM KCl
250 mM KCl
WT
seip-1(tm4221)
10 µm
e
DAPI
DAPI / DIC
WT
seip-1(tm4221)
10 µm

Fig. S1. ***seip-1(tm4221)*** **mutants have permeable eggshells.**

(a) Time-lapse confocal images of wild-type and *seip-1(tm4221)* embryos at the early cleavage stage. Plasma membranes were labelled by GFP::PH(PLC1delta1) and nuclei were labelled by mCherry::Histone. The relative time points are shown at the top left. White arrows indicate that cytokinesis proceeds more slowly and aborts halfway. Yellow arrows indicate cell fusion mediated by abrupt disruption of the adjoining plasma membrane.

(b) Diagram of the composition of the *C. elegans* eggshell. Six eggshell layers are distinguished by different colors outside the plasma membrane. The first and second polar bodies are located in the perivitelline space and periembryonic space, respectively.

(c) Transmission electron microscopy images of wild-type and *seip-1(tm4221)* eggshells. The eggshell layers are distinguished by colors according to B.

(d) Differential interference contrast microscopy (DIC) images of wild-type and *seip-1(tm4221)* embryos which were soaked in hypotonic (100 mM KCl) or hypertonic (250 mM KCl) solution.

(e) Fluorescence and DIC images of wild-type and *seip-1(tm4221)* embryos which were stained with DAPI.

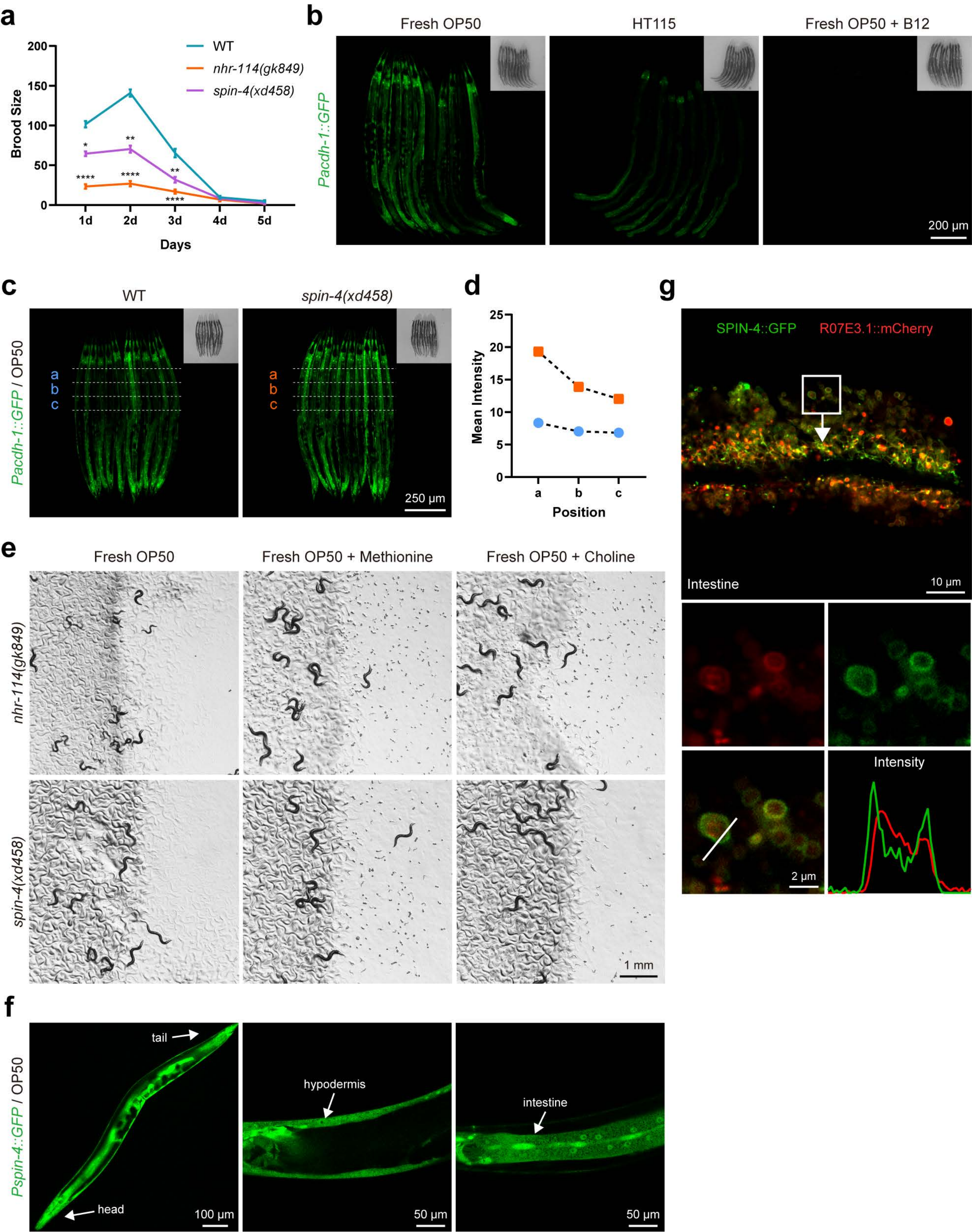

a
WT
nhr-114(gk849)
spin-4(xd458)
Brood Size
Days
1d
2d
3d
4d
5d
b
Fresh OP50
HT115
Fresh OP50 + B12
Pacdh-1::GFP
200 μm
c
WT
spin-4(xd458)
Pacdh-1::GFP / OP50
250 μm
d
Mean Intensity
Position
e
Fresh OP50
Fresh OP50 + Methionine
Fresh OP50 + Choline
nhr-114(gk849)
spin-4(xd458)
1 mm
f
Pspin-4::GFP / OP50
tail
head
hypodermis
intestine
100 μm
50 μm
50 μm
g
SPIN-4::GFP
R07E3.1::mCherry
Intestine
10 μm
2 μm
Intensity

Fig. S2. The sterility of *nhr-114* and *spin-4* mutants is diet-dependent.

(a) Quantification of brood size of wild type, *nhr-114(gk849)* mutants and *spin-4(xd458)* mutants over five time periods from the beginning of adulthood. Error bars represent SEM. The numbers of tested animals for wild type, *nhr-114(gk849)* and *spin-4(xd458)* are 13, 20 and 18, respectively. Statistically significant differences between wild type and each mutant at each time point were determined by nonparametric test (Kruskal-Wallis H-test) with *post hoc* Dunn's test: *P < 0.05, **P < 0.01, ****P < 0.0001.

(b) Confocal images of *Pacdh-1::GFP* reporter animals fed on different diets with or without B12 supplement. Insets are bright-field images.

(c) Confocal images of the *Pacdh-1::GFP* reporter in the wild-type or *spin-4(xd458)* background. Insets are bright-field images. Three regions of each image were segmented to calculate the mean fluorescence intensity.

(d) Quantification of fluorescence intensity at the three different positions in C.

(e) Bright-field images of worm plates carrying *nhr-114(gk849)* or *spin-4(xd458)* mutants on fresh OP50 supplied with or without 10 mM methionine or 50 mM choline.

(f) Confocal images of adult *Pspin-4::GFP* animals. The fluorescence of *Pspin-4::GFP* is observed throughout the worm, including head, tail, hypodermis and intestine, as indicated by the white arrows.

(g) Confocal images of adult animals expressing SPIN-4::GFP and mCherry::R07E3.1. The enlarged images of the boxed area show that the fluorescence of SPIN-4::GFP encircles the signal from the mCherry-tagged lysosomal peptidase R07E3.1. The line profile of the signals is shown at the bottom right.

Supplementary Table S1. *C. elegans* strains used in this study.

Strain	Source	Genotype
N2	CGC	wild type (Bristol)
CB4856	CGC	wild type (Hawaiian)
XD4613	NBRP(Japan)	*seip-1(tm4221) V*
XD3546	Huang Lab	*seip-1(tm4221) V; xdEx1640 [Pseip-1::seip-1 + rol-6(su1006)]*
RT130	CGC	*pwIs23 [vit-2::GFP]*
XD5440	Huang Lab	*seip-1(tm4221) V; pwIs23 [vit-2::GFP]*
JIM113	CGC	*unc-119(ed3) III; ujIs113 [Ppie-1::mCherry::H2B + Pnhr-2::mCherry::HIS-24 + unc-119(+)] II*
OD58	CGC	*unc-119(ed3) ltIs38 [Ppie-1::GFP::PH(PLC1delta1) + unc-119(+)] III*
XD4901	Huang Lab	*ujIs113 [Ppie-1::mCherry::H2B + Pnhr-2::mCherry::HIS-24 + unc-119(+)] II; ltIs38 [Ppie-1::GFP::PH(PLC1delta1) + unc-119(+)] III*
XD4866	Huang Lab	*seip-1(tm4221) V; ujIs113 [Ppie-1::mCherry::H2B + Pnhr-2::mCherry::HIS-24 + unc-119(+)] II; ltIs38 [Ppie-1::GFP::PH(PLC1delta1) + unc-119(+)] III*
XD1017	Huang Lab	*seip-1(tm4221) III/nT1 [qIs51] (IV;V)*
XD3714	Huang Lab	*seip-1(tm4221) V; spin-4(xd286) III*
XD3715	Huang Lab	*seip-1(tm4221) nhr-114(xd287) V*
XD5260	Huang Lab	*spin-4(xd458) III*
XD5303	Huang Lab	*seip-1(tm4221) V; spin-4(xd458) ltIs38 [Ppie-1::GFP::PH(PLC1delta1) + unc-119(+)] III*
XD4860	Huang Lab	*seip-1(tm4221) nhr-114(xd428) V*
XD6124	Huang Lab	*seip-1(tm4221) nhr-114(xd428) V; ltIs38 [Ppie-1::GFP::PH(PLC1delta1) + unc-119(+)] III*
VC1760	CGC	*nhr-114(gk849) V*
XD5905	Huang Lab	*spin-4(xd458) ltIs38 [Ppie-1::GFP::PH(PLC1delta1) + unc-119(+)] III*
XD6122	Huang Lab	*nhr-114(gk849) V; ltIs38 [Ppie-1::GFP::PH(PLC1delta1) + unc-119(+)] III*
RB1434	CGC	*mmcm-1(ok1637) III*
RB755	CGC	*metr-1(ok521) II*
QC122	CGC	*paqr-2(tm3410) III; pcyt-1(et9) X*
XD6503	Huang Lab	*pcyt-1(et9) X; ujIs113 [Ppie-1::mCherry::H2B + Pnhr-2::mCherry::HIS-24 + unc-119(+)] II*
VL749	CGC	*wwIs24 [Pacdh-1::GFP + unc-119(+)]*
XD6333	Huang Lab	*spin-4(xd458) III; wwIs24 [Pacdh-1::GFP + unc-119(+)]*
XD5403	Huang Lab	*xdEx2409 [Pspin-4::GFP + Podr-1::RFP]*
XW7951	Xiaochen Wang	*qxIs448 [R07E3.1::mCherry]*
XD5553	Huang Lab	*qxIs448 [R07E3.1::mCherry]; xdEx2483 [Pvha-6::SPIN-4::GFP + Podr-1::RFP]*
XD6500	Huang Lab	*seip-1(tm4221) V; mmcm-1(ok1637) III*
XD6502	Huang Lab	*seip-1(tm4221) V; metr-1(ok521) II*
XD6504	Huang Lab	*seip-1(tm4221) V; pcyt-1(et9) X; ujIs113 [Ppie-1::mCherry::H2B + Pnhr-2::mCherry::HIS-24 + unc-119(+)] II*
XD6339	Huang Lab	*seip-1(tm4221) V; wwIs24 [Pacdh-1::GFP + unc-119(+)]*
BX30	CGC	*fat-3(wa22) IV*
XD6294	Huang Lab	*seip-1(tm4221) V; fat-3(wa22) IV; ltIs38 [Ppie-1::GFP::PH(PLC1delta1) + unc-119(+)] III*
XD6295	Huang Lab	*seip-1(tm4221) V; spin-4(xd458) III; fat-3(wa22) IV*
XD6296	Huang Lab	*seip-1(tm4221) nhr-114(xd428) V; fat-3(wa22) IV; ltIs38 [Ppie-1::GFP::PH(PLC1delta1) + unc-119(+)] III*
